# How Boys Are Made Bad

**Published:** 1877-10

**Authors:** Ben. H. Pratt


### THE BISTOURY.

Vol. XIII. ELMIRA, N. Y., OCT., 1877. No. 3.

### Contributions.

### HOW BOYS ARE MADE BAD.

BEN. H. PRATT.

How often we hear it remarked, of a dissipated and dissolute young man, that he is going to the devil through pure perversity; that everything has been done for him that a good home and kind parents could do; that his training, education, home influence and family associations have been of the very best that could be had, and yet he has turned out bad in spite of them all. Indeed, in the case of those whose surroundings seem to have been of the most elevating and cultivating and ennobling sort, the results are almost invariably the opposite of what one would be led to expect. Ministers' sons are proverbially the worst boys in the community, and so generally do they turn out scape-graces that it has now become a matter of considerable surprise and wonderment when the son of a clergyman or of a staid deacon exhibits symptoms of steadiness and grows up without becoming a notorious scamp or a loaferish non-entity. It has come to this—that we don't expect much from the sons of the good men of the community, and it is very rare to find a boy who follows in the footsteps of his father. The wise men and the great men are forced to find their sons drifting into nobodies, or worse, and the dependence of the nation seems to be in the children of the less cultivated. Out of the best families come the scallawags of the community, and from the homes of the lowly spring the brightest intellects and the steady, sober, useful young men. There are brilliant exceptions, of course, but just enough to prove the rule.

Now these are phenomena which seem incomprehensible to most people, and especially poignant is the grief of good fathers and mothers over the waywardness of their loved sons, upon whose training they have expended their best endeavors, and whose prayers for the regeneration of their loved ones have almost hourly been addressed to the throne of grace, and yet without any apparent response in the way of an amended life. They daily note, with the deepest chagrin, the slow but sure advancement of young men to positions of trust and honor and popular admiration, while their own sons are gradually being lost sight of in the life-journey, seeming to have no lot with the deserving, and no title to the community's praise.

They cannot comprehend the reason for all this. They do not see why the education, culture and refinement with which they have labored so hard to endow their children should go for naught, while those young men, who have had no advantages aside from those which their own efforts have achieved, become the superiors of the sons of wealth and refinement in every department of business, professional or other.

We believe we can ravel this mystery, and point out the precise cause of all this trouble, and in doing so, are obliged to say that the difficulty lies in the sad mistake of parents; first, in not providing home attractions for their children; second, in not compelling their children to depend upon themselves.

What sort of a home does the average minister or deacon or orthodox church member provide for his children? It is a home of comfort, of course, and usually one of elegance in a greater or less degree. There is plenty there—plenty to eat, to wear, to read—plenty of incentive to luxurious ease—plenty to teach the youth to depend upon his father—plenty of servants to wait upon him—plenty of leisure to beget laziness—plenty of sympathy to spoil the family pet—plenty of everything except what the young man needs to make him self-reliant. Perhaps even this condition of affairs would not spoil a young man if his recreation was as judiciously consulted as are his other whims. What are the recreations provided in the average home of the staid, for the children in the family? Books, of course—good books, such as the wise enjoy—theological, historical, biographical, poetical, and now and then a romance by an eminent clergyman, or even a novel or two with a deep moral tone, a sepulchral tone, as it were, and as grim as the joke of a clown in the valley of Jehosaphat. Newspapers, certainly—denominational, educational, ably edited, but not especially attractive to the youth. His eye runs up and down their columns for something in the way of an anecdote or a facetious allusion, but seldom successfully. Games, limited—checkers, authors, buzby, croquet, parchesi, dominoes, rarely backgammon, because there are dice which gamblers sometimes use. Music, piano—takes ten years to learn to play it, to say nothing of the tedious hours of practice, the scolding of preceptors, and the eternal tumpetum of scales. This is home, but it's the home of those who have become staid and settled down into the proprieties. It's not a boy's home. With all this, the natural boy, sitting on a velvet ottoman, with his nose flattened against the plate glass of his father's window, looks out and envies the ragamuffin who is wading bare-foot in the gutter, or cracking stolen walnuts on the curbstone. Bigger-grown, he finds the literature in the hotel reading-room a fascination—cards are a charming novelty to him—billiards delight him—the dance enraptures him—he finds jolly companions who have homes like his, just as stupid and just as gloomy—they are like colts let out to pasture, and they kick high, run mad with joy over their new found liberty—they go to the devil, and they don't often come back. Why do they see such a fascination in bad associations? Not because they like the bad better than the good; not because they are naturally bad themselves; but because that which they have not been allowed at home is so great a novelty that they are carried away with it, as a child with a new toy. There's a glamour of brilliancy thrown around the novelties they meet with when they get out of the parental sight that is too strong for their weak powers of resistance, and they are drawn within the charmed circle as by a spell. The contrast with the home is so marked that the family circle becomes a bore, and the quiet household a sort of penitential gloom, out of which they get with haste, scarcely enduring a stay for meals and lodging. This is what makes boys bad.

Now there is a home which, though it may not boast the splendor of the saloon, is quite as attractive to the young. It is the home where children are allowed many of the amusements which the saloon offers. It is the home where brothers and sisters and neighbors are allowed to indulge in all amusements that are in themselves innocent—where card playing is not only allowed by the parents, but is taught by them—where dancing is never prohibited, but encouraged as a healthful pastime and a cultivator of graceful movement and polite deportment—where the piano is no more respectable than the violin, the flute, the guitar, or even the banjo—where a good joke and a hearty laugh are welcomed by old and young—where a good story isn't taboed because it is a novel—where instruction is combined with amusement, and piety is not inconsistent with fun—where no shock is felt when the father of the family, at ten o'clock, invites the quartette from the euchre or whist table to join him in family worship. This is the sort of home that holds our young men—and our young women, too—within the sphere of good influences, and prevents young people from running to the saloon and the dance house for their amusements. There is much wisdom in the scriptural quotation: "Be ye wise as serpents and harmless as doves," and parents cannot con this saying too closely. There is a wisdom which is folly, and there is a folly which tends to wisdom. We have watched the sons of the strict and the sons of the liberal, and rarely have we found the former turn out well, and as rarely, in our experience have the latter turned out ill.

Again, boys are often allowed to occupy a place in the family which no member of it can accept without compromising himself; that is, he is assigned no active part in the household. Every member of the family should bear the same relation to it that a partner bears to a business firm. He should have some regular duty to perform, one from which nothing but illness might excuse him, and this position should be no sinecure, either. Something to do should be sought for him, and it should be something necessary to be done, and not a trumped up work simply to afford employment. This will early teach boys, first, what work is; second, that they are of some use; third, to become in a measure self-dependent, since they earn some part of what they receive; fourth, to acquire habits of industry. A boy's work should not only be constant, but punctually performed every day, and the manner of its performance should be carefully taught. Thus neatness, skill and handiness are inculcated. It is surprising how soon a boy will grow into a habit of doing something if he knows he must. "My chores" are the means of keeping many a boy out of a scrape. We always fear for the boy that has no chores to do. It is not possible that any family cannot find work for every child in it, and it would be exceedingly foolish in us to attempt to define what work the child may be put to. But there is a great mistake made by some parents in requiring work from their children and not defining that work. The parent who keeps a boy dancing attendance continually upon his or her whims—requiring him "to be around all the time in case he's wanted"—is a tyrant. Such service takes all the tuck out of the best boy in the world, and makes a slave of him. Every boy should have his play spells as well as his work spells and study spells. All work and no play is as bad as all play and no work. The twenty-four hours of a boy's day should be judiciously apportioned off into hours of sleep, study, work and play, and thus he will grow up balanced, and not humped either as to back or brain.

Parents don't think enough about these things. They are apt to act upon the impulse of the moment, forbidding one day what they allow the next—now compelling to much restraint—now allowing too large a liberty. Some reason too much with their children and others never deign to give a reason for anything. A policy of government is as necessary in the family as in the State. Arbitrary and changeful laws would be a curse to the State, and they are such in the family. Let the child know what to expect, and never disappoint him, whether it's encouragement or a thrashing. It's the loose way we govern our children that makes them turn out bad. Neglect of their coming and going and doing and being is as bad as a constant supervision of their every act. The one makes them indifferent as to what is to become of them, and the other makes them eye-servers, and begets cunning and trickery.

We believe that the average boy is inclined to be a something rather than a nothing, and if he turns out the latter it is rather the fault of the parent than of the boy. Boys can't be *made* good, but they can be kept from becoming bad. Boys are oftener *driven* to evil than *pulled* into it, and one injudicious parent does more to make boys bad than ten scapegrace companions do.

WOMEN PHYSICIANS IN INDIA.—An English lady, Lady Anna Gore-Langton, writing from India, says:—"Nothing is more painful than to see the vacant, hopeless, melancholy faces of the adult women; and nothing is more wanted than lady doctors, who might save Indian women much suffering. Sir Salar Jung exerted himself some time back to secure a lady doctor for India. He had to send to America for one, and she has now a large practice among the native women."

